# Multiple endocrine neoplasia type 1: a new germline “homozygous” variant (c.201delC) caused by detection errors

**DOI:** 10.1186/s13053-022-00216-2

**Published:** 2022-03-07

**Authors:** Fan Zhang, Xiaohui Yu, Xiaoli Wang, Hua Shao

**Affiliations:** 1 Department of Hematology, Shengjing Hospital of China Medical University, Shenyang, China; 2 Department of Endocrinology and Metabolism, Institute of Endocrinology, NHC Key Laboratory of Diagnosis and Treatment of Thyroid Diseases, The First Hospital of China Medical University, 110001 Shenyang, Liaoning Province P. R. China; 3 Department of General Surgery, Shengjing Hospital of China Medical University, Shenyang, China

**Keywords:** Multiple endocrine neoplasia type 1, *MEN1*, Germline variant, Sanger sequencing, Exon

## Abstract

**Background:**

Multiple endocrine neoplasia type 1 (MEN1) is a hereditary cancer syndrome caused by germline variants in the *MEN1* gene located on chromosome 11q13. We found a Chinese woman who had a pancreatic tumor, parathyroid tumor, adrenal tumor, and suspicion of gastrinoma.

**Case presentation:**

The proband and her immediate family members underwent genetic detection. The results showed that two of the proband’s six relatives had the same variants as the proband, and her sister also had the typical symptoms of MEN1. However, the first- and second-time genetic detection results showed that they were homozygous variants, which did not conform to Mendelian inheritance laws. Multiplex ligation-dependent probe amplification (MLPA) was used to rule out homozygous variants caused by a deletion of gene fragments in the proband and her immediate family members. The MLPA results showed that the gene deletion was absent in the *MEN1*. The results from the third genetic detection (redesigned the primer) showed that they had a heterozygous variant. A new *MEN1* germline variant [c.201delC (p.Ala68Profs*51)], which could induce MEN1, was found in this study.

**Conclusions:**

This newly identified germline variant could improve the identification of clinical phenotypes and the early diagnosis of MEN1. Clinician should consider the present of situation that intron variant causing detection error. Re-designing the primers close to the variant site for gene detection could avoid this situation.

**Supplementary Information:**

The online version contains supplementary material available at 10.1186/s13053-022-00216-2.

## Background

Multiple endocrine neoplasia type 1 (MEN1), characterized by neoplasia in multiple endocrines and nonendocrine tissues, is an autosomal dominant hereditary tumor syndrome with a prevalence of about 1/30,000. The three major endocrine tissues affected by tumors in MEN1 are the parathyroid (95%), enteropancreatic neuroendocrine (50%), and anterior pituitary (40%). A diagnosis of MEN1 may be established in an individual by one of three criteria: on the basis of the occurrence of two or more primary MEN1-associated endocrine tumors (i.e. parathyroid adenoma, enteropancreatic tumor, and pituitary adenoma); the occurrence of one of the MEN1-associated tumors in a first-degree relative of a patient with a clinical diagnosis of MEN; and identification of a germline MEN1 mutation in an individual, even if they are asymptomatic and have not yet developed serum biochemical or radiological abnormalities indicative of tumor development [1]. Besides, MEN1 patients could present with many other hormone-secreting, hormone nonsecreting, and nonendocrine tumors, such as adrenal cortical tumors, foregut carcinoids (bronchial, thymic or of the gastric enterochromaffin-like cells), facial angiofibromas, truncal collagenomas, lipomas, meningiomas, Barrett’s esophagus, leiomyoma (uterine in females or in the esophagus), and ependymoma [2]. Approximately 25% of MEN1 patients die of a malignant gastrinoma (gastrointestinal neuroendocrine tumors) or foregut carcinoid tumor [2].

The related gene *MEN1*, which encodes the protein MENIN [3, 4], was first reported in 1988 [5] and is located on chromosome region 11q13 [3, 4]. It is composed of 10 exons that encode a 610 or 615 amino acid nuclear protein. MENIN interacts with the activator protein 1 (AP1) transcription factor JunD to inhibit JunD-activated transcription [6], which is related to cell growth regulation, cell cycle, genome stability, and synaptic plasticity. Detection of *MEN1* gene variants helps to distinguish MEN1 syndrome from other solitary tumors, such as somatic loss of heterozygosity (LOH) on chromosome 11q13, which is observed in 5–50% of such non-hereditary common sporadic tumors [7]. *MEN1* gene variants are distributed throughout the coding region, which has no obvious hot spots and no obvious genotype/phenotype correlation with tumor spectrum or clinical characteristics. More than 1,000 variants have been reported, including frameshift deletions or insertions (40%), nonsense (20%) and missense (25%) variants, splice site changes (8%), deletions or insertions (6%), and large deletions (1%) [7]. Thus, 70–75% of the *MEN1* gene variants are inactivating, causing premature protein truncation. However, many variants (such as rare missense in nonfamilial cases) can be classified as variants of unknown clinical significance due to the lack of available assays that could provide evidence for an adverse physiological consequence of such variants [8].

In this study, Sanger sequencing was performed on the proband who had a pancreatic tumor, parathyroid tumor, adrenal tumor, and suspicion of gastrinoma. The suspected *MEN1* variant site in exon 2 was screened using the combination of bioinformatics and public database. The results indicated that a variant in an intron could affect the sequencing results and led to the occurrence, at first, of homozygotes. The three family members who were detected as homozygotes in the 1st and 2nd times, were found to be heterozygotes by resequencing. A new *MEN1* germline variant [NM_130802: c.201delC (p.Ala68Profs*51) on Chr11:64577381 on assembly GRCh37] was found, which was the genetic pathogenesis of MEN1 in this family.

## Case presentation

### Patients

The proband (II-3) was a 49-year-old woman who was born to a family native to Liaozhong, Liaoning Province of China. She was admitted to our hospital with a “3-year duration of large appetite and rapid hungering, intermittent recurrent fatigue, and palpitations, sweating for 2 years.” Denial of family history of illness.

A series of examinations was performed as below:

### Pancreas-related results

Islet cell tumors are the most common endocrine gland tumor in MEN1. During hospitalization, the proband had the typical Whipple’s triad. She had two episodes of fasting hypoglycemia (2.63 mmol / L and 1.72 mmol / L, respectively). The proband’s symptoms were quickly relieved by eating or receiving a glucose supplement. Meanwhile, the proband’s serum glucose, insulin (INI), C-peptide (CPS), and pro-insulin (pro-INS) levels were tested, and the results showed that glucose was decreased while the insulin level was either in the normal reference range or increased at the time of the hypoglycemia (Supplementary Table 1). The insulin release indexes were 0.55 and 0.52 (normal range < 0.3) when the hypoglycemia occurred. A prolonged oral glucose tolerance test (OGTT) was performed, and the results presented hypoglycemia at the 240th and 300th minute with glucose levels of 2.01 mmol/L and 1.95 mmol/L, respectively. The corresponding insulin release indexes were 0.48 and 0.43, respectively (Supplementary Table 2).

**Fig. 1:**
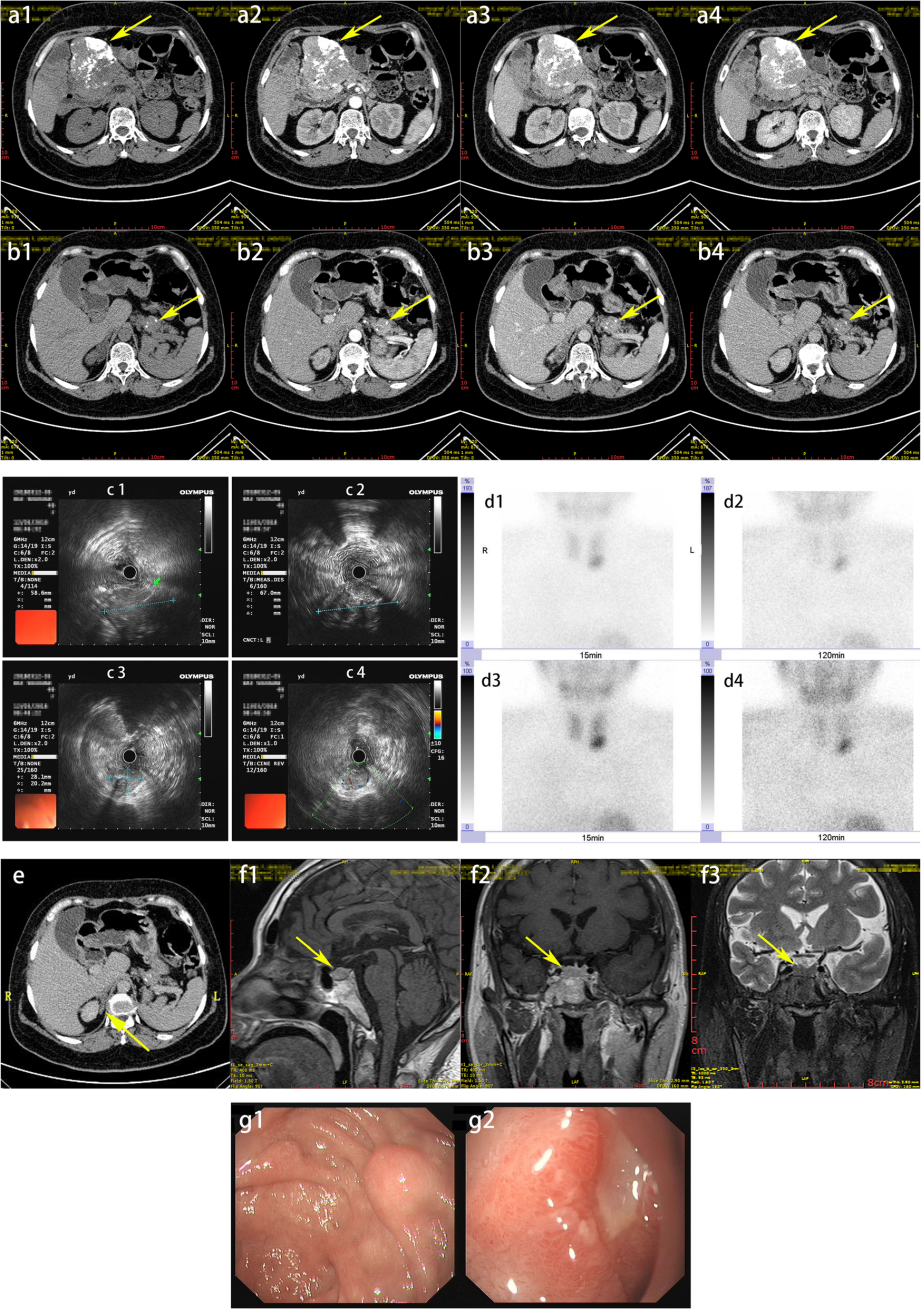
The proband’s clinical imageology examination images. **A** a1–a4: Pancreatic neck lesions (8.0 × 6.8 × 8.4 cm) (a1, plain scan; a2, arterial phase; a3, portal phase; a4, delayed phase). **B** b1–b4: Ring enhanced nodule at the tail of the pancreas (2.8 × 2.7 cm) (b1, plain scan; b2, arterial phase; b3, portal phase; b4, delayed phase). **C** The hypoechoic mass and the internal echoes were mixed. c1, c2: head of the pancreas (cannot be observed in the full field); c3, c4: the tail of the pancreas (2.81 × 2.02 cm). **D** d1, d3: 15 min after the 99mTc-stabimidine (MIBI) injection, an area of increased imaging agent appeared in the lower lobe of the left thyroid; d2, d4: 2 h after the injection, the area of distribution of the imaging agent under the left lobe of the thyroid did not decrease significantly. **E** Abdominal CT result showing that a mass occupied the right adrenal gland. **F** f1–f3. Pituitary-enhanced MRI examination showing that the hypophysis was full in shape (height 0.95 cm). f1, f2: T1WI; f3: T2WI. f1: sagittal view; f2, f3: coronal view. **G** g1, Multiple submucosal uplifts (the larger was about 0.6 × 0.8 cm) at the junction of the duodenal bulb and the descending part of the duodenum; g2, the distal shallow ulcer in the descending part of the duodenum was about 1.0 × 0.5 cm in size

The imaging examinations provided us with more information. The CT results of the pancreas showed that the mass in the pancreatic neck (8.0 × 6.8 × 8.4 cm) could have been a solid pseudopapillary tumor (Fig. 1 a1–a4). Circular reinforced nodules in the tail of the pancreas (2.8 × 2.7 cm) (Fig. 1 b1–b4) were considered to be homologous with the pancreatic neck mass (solid pseudopapillary tumor). The arterial phase of the pancreatic tail artery significantly strengthened the small nodules at the early stage, which could have been a neuroendocrine tumor. EUS is considered to be the most sensitive single method for the detection of pancreatoduodenal tumors. Compared with a CT scan and somatostatin receptor imaging, EUS has obvious advantages [9]. The patient’s ultrasound gastroscopy showed that the head of the pancreas could not be observed in the full field because the mass was too big (Fig. 1 c1–c2). The mass in the tail of the pancreas (2.81 × 2.02 cm) (Fig. 1 c3–c4) was a hypoechoic mass, and the internal echoes were mixed.

Therefore, according to the laboratory and imaging examination, the mass in the pancreas was likely an islet cell tumor. However, the proband refused invasive examinations (including CT or ultrasound-guided needle biopsy), leading to a lack of pathological results. Later on, we checked other endocrine glands of the proband.

### Parathyroid-related results

In MEN1 patients, the prevalence of parathyroid tumors is about 95% [8]. The proband’s serum tests showed that the serum calcium (Ca^2+^) was increased (2.47–2.74 mmol/L, normal range 2.17–2.57 mmol/L) and serum phosphorus (P) was decreased (0.71–0.96 mmol/L, normal range 0.81–1.52 mmol/L). The serum parathyroid hormone was 553.6 pg/mL (normal range 15–65 pg/mL).

The results of the patient’s bone mineral density measurements presented a decrease in bone mass in the lumbar spine and hip joint bones. Imaging modalities, such as parathyroid ultrasound and ECT, are valuable in MEN1 patients [10]. Parathyroid ultrasound showed a low echo at the posterior margin of the thyroid (right, 7.9 × 4.5 mm; left, 11.0 × 3.6 mm). Fifteen minutes after the 99mTc-stabimidine (MIBI) injection, the parathyroid ECT demonstrated an area of increased imaging agent in the lower lobe of the left thyroid (Fig. 1 d1, d3). Two hours after the injection, the imaging agent that was increased in the left lobe of the thyroid had not significantly decreased (Fig. 1 d2, d4), but no obvious abnormality on the right side. Therefore, the hypoechoic right parathyroid gland reported by ultrasound may be caused by hypertrophic parathyroid gland. This phenomenon was caused by a highly functional parathyroid adenoma.

The above results confirmed that the proband had primary hyperparathyroidism that could derive from the highly functional parathyroid adenoma.

### Adrenal gland-related results

Asymptomatic adrenal tumors occur in about 20–73% of MEN1 patients. Patients with MEN1 tend to develop aldosterone and adrenocortical cancer but are less likely to develop pheochromocytoma than those with adrenal incidentaloma [11]. The proband’s CT result showed that a mass occupied the right adrenal gland (Fig. 1E).

The biochemical evaluation of adrenal tumors includes an evaluation of plasma free cortisone (COR), evening cortisol, plasma renin, and aldosterone concentration, and 1-mg overnight dexamethasone (DXM) suppression test [10]. In this case, the basal COR rhythm was turbulent. The 1-mg overnight DXM suppression test results demonstrated that the inhibition rate of COR was 76.4% (normal range > 50%). Therefore, Cushing syndrome was excluded. The plasma renin, aldosterone and sex hormones concentrations were normal. Moreover, the patient did not have hypertension.

All of these findings suggested that a nonfunctional adrenal tumor existed.

### Pituitary-related results

The serum prolactin (PRL) was two times the normal upper limit (1253 mIU/L, normal range 40–530 mIU/L; rechecked 1395 mIU/L). Serial basal PRL levels greater than 4240 mIU/L confirmed a prolactinoma [10]. The results of the metoclopramide stimulation test showed that PRL was 1.88 times higher than the basic value and the inhibition rate of PRL was 60.7% (normal range > 50%), as found by the bromocriptine inhibition test. This indicated that the patient suffered from hyperprolactinemia. Other pituitary hormones, including growth hormone (GH), IGF-1 (insulin-like growth factor 1), luteinizing hormone (LH), follicle-stimulating hormone (FSH), thyroid-stimulating hormone (TSH), and free-thyroxine (FT4), were normal.

An MRI of the pituitary gland with contrast is the best diagnostic imaging [10]. The pituitary-enhanced MRI examination showed that the hypophysis was full in shape (height 0.95 cm) (Fig. 1 f1–f3), but no definite tumor was found. Based on the above serum and imaging examination, we believe that the proband’s hyperprolactinemia could have been due to hyperplastic pituitary cells.

### Stomach and duodenum-related tumor

The incidence of neuroendocrine tumors (NETs) in gastrointestinal and pancreatic tissues is about 30–75%. However, autopsy studies have shown that the prevalence is as high as 80–100% [12]. NETs without function are more common than functional tumors. Moreover, because approximately 25% of MEN1 patients die from cancer due to malignant gastrinomas [2], an examination of the gastroduodenum is necessary. Serum pepsinogen I and gastrin 17 (GAS-17) were increased significantly (547 µg/L, normal range 70–150 µg/L; 78.6 pmol/L, normal range 0.01–5 pmol/L, respectively). The antibody result for *Helicobacter pylori* infection was negative.

Gastroscopy showed multiple submucosal uplifts (the largest was about 0.6 × 0.8 cm) at the junction of the duodenal bulb and the descending part of the duodenum (Fig. 1 g1). A distal shallow ulcer in the descending part of the duodenum was about 1.0 × 0.5 cm in size (Fig. 1 g2). The pathological diagnosis was moderate inflammation and erosion of the descending part of the duodenum. Duodenal gastrinomas in MEN1 syndrome are usually small (smaller than 1 cm), multifocal, and occur mainly in the proximal duodenum [10]. Therefore, no obvious gastrinoma lesions were found during the gastroscopy.

Known as Zollinger-Ellison syndrome (ZES), peptic ulcer disease can be caused by functioning NET-secreting gastrin because of increased gastric acid secretion [10]. As a result of the increased level of gastrin 17 and the occurrence of duodenal ulcers, the proband was likely to have had gastrinomas.

The main diagnosis of the proband was MEN1. She had pancreatic insulinoma, a tumor of the adrenal gland, primary hyperparathyroidism (adenoma of the parathyroid), hyperprolactinemia, gastric and duodenal ulcers (possibly Zollinger-Ellison syndrome), a right renal cyst, and myoma of the uterus.

**Fig. 2:**
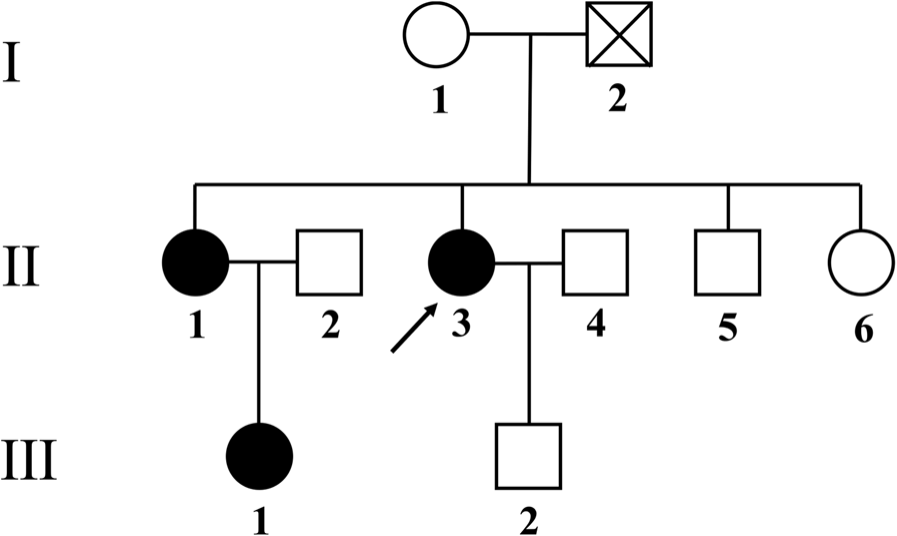
Pedigree of the proband. The patient’s family consisted of three generations, including 10 members. The proband’s father (I-2) of the first generation was deceased. Included the proband and her immediate family members, seven people totally were tested (I-1, II-1, II-3, II-5, II-6, III-1, III-2), and the three members who carried the gene variants were the proband (II-3), the proband’s sister (II-1), and the proband’s niece (III-1)

It is well known that MEN1 is an autosomal dominant genetic disease. Therefore, in addition to the proband, we also carried out genetic tests on her relatives. The patient’s family consisted of three generations, including ten members. The proband’s father (I-2) of the first generation was deceased. Included the proband and her immediate family members, seven people totally were tested (I-1, II-1, II-3, II-5, II-6, III-1, III-2) (Fig. 2). Three members carried gene variants, including the proband (II-3), the proband’s sister (II-1), and the proband’s niece (III-1). The results of the other relatives’ genetic tests indicated normal *MEN1* gene sequences. No close relatives among the members of this family had married. The genetic map of the family members is given in Fig. 2. All family members in this study provided signed informed consent.

### Sanger sequencing

In 90% of cases, MEN1 is usually inherited from the affected parent; the other 10% of cases occur by de novo variant. Germline inactivating variants of the *MEN1* gene on chromosome 11 is the main cause of MEN1 [10]. To determine whether the gene had changed, the proband and six relatives had Sanger sequencing and bioinformatics analyses (the methods of gene detection are detailed in the supplementary file).

The gene detection results of the first times experiment (1st seq) showed that the proband (II-3), her sister (II-1), and her niece (III-1) had c.201delC change of NM_130802 in exon 2, which caused amino acid changes (p.Ala68Profs*51) that might lead to MEN1. Interestingly, the genetic changes were homozygous variants (Fig. 1 and 1st times detection). No genetic variants were found in the other relatives. However, we analyzed the patient’s family pedigree, and neither the mother (I-1) nor the son (III-2) of the proband had this genetic variant. This result was not consistent with Mendelian genetic law. Therefore, we commissioned the other technicians to redesign the primers (for 2nd seq). We retested the samples and got the same result (c.201delC) (Fig. 2 and 2nd time detection).

**Fig. 3:**
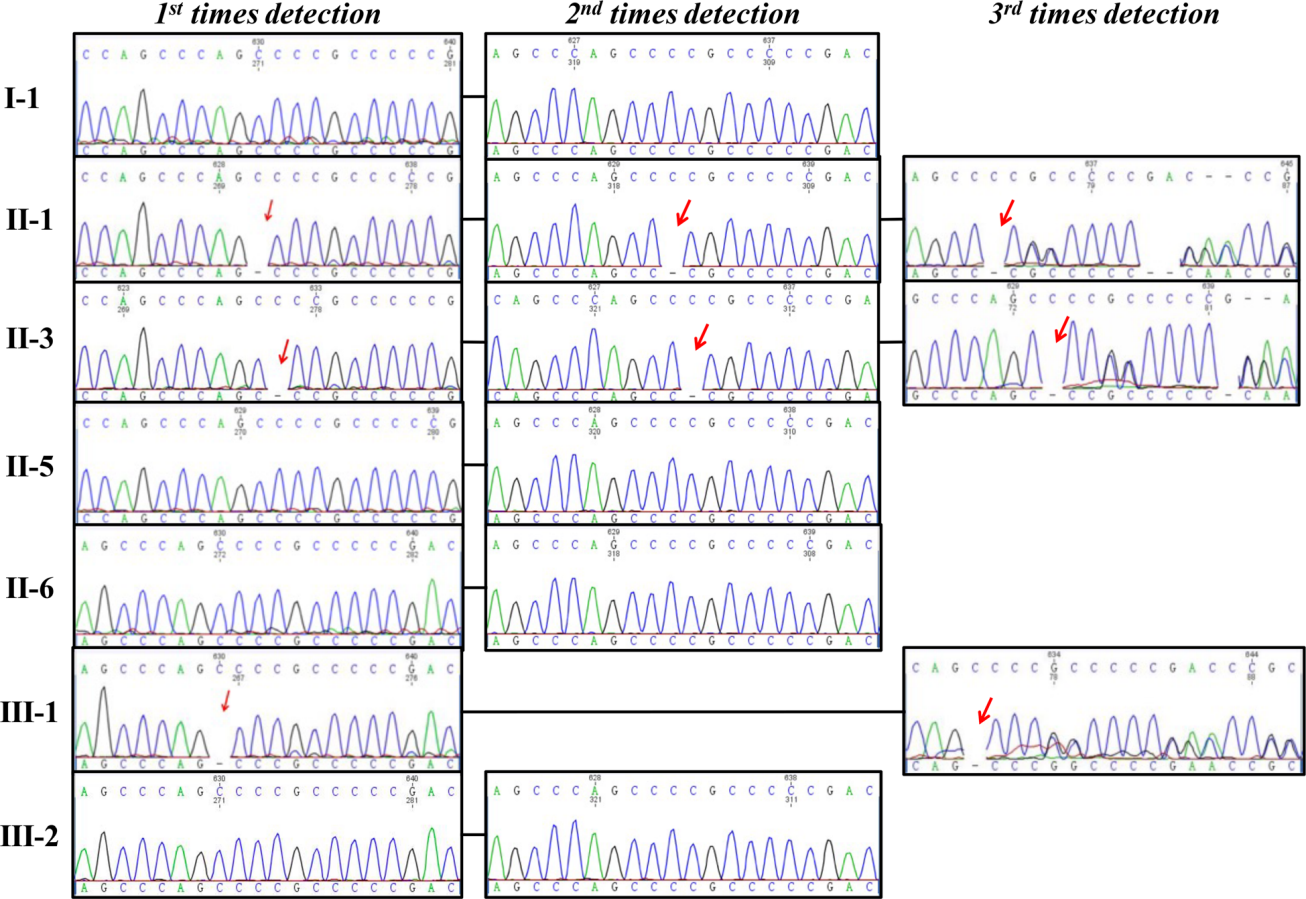
Sanger sequencing results. The c.201delC change of NM_130802 in exon 2, which caused amino acid changes (p.Ala68Profs*51) that may have led to MEN1. The genetic changes were heterozygous variants (3rd times detection), but was wrongly detected as homozygous variants (1st, 2nd times detection)

Beijers et al. [13] found a germline and somatic mosaicism in a family with MEN1. They analyzed the blood using MLPA, which showed a minimal but consistently decreased signal for the MEN1-specific MLPA probes. The results from the MLPA trials on the proband (II-3) and her son (III-2), her sister (II-1), and her niece (III-1) were normal. This demonstrated that the gene deletion was absent in the *MEN1* of this family (Supplementary Fig. 1).

A Chinese family presenting with MEN1, with a heterozygous variant c.825-1G > A and a variant in intron 5 (IVS 5 − 1 G > A) of the *MEN1* gene, was considered by Zhiwei Ning et al. [14]. Considering the possibility of an intron change, we redesigned the primer (for 3rd seq) in the middle of exon 2 and redetected. It turned out that the proband, her sister, and her niece had heterozygous genetic variants (c.201delC, p.Ala68Profs*51) in chromosome 11 (chr11:64577381), which is consistent with Mendelian genetic law (Fig. 3 3rd times detection).

## Discussion and Conclusions

In this study, we found a proband with typical tumor symptoms of MEN1, including a pancreatic tumor, parathyroid tumor, adrenal tumor, and suspicion of gastrinoma, as well as pituitary changes. Subsequently, Sanger sequencing was used to test the proband for the *MEN1* gene. We found a new gene variant in exon 2 of *MEN1*, which could lead to these clinical symptoms. Due to the heritability of MEN1, the proband’s family members were tested as well. Subsequently, we found that this Chinese family had MEN1 and we found a new germline variant of NM_130802 [c.201delC (p.Ala68Profs*51) on Chr11:64577381 on assembly GRCh37].

Because of the results from the genetic testing, we recommended that the proband’s family members be hospitalized for screening for disease related to MEN1. The proband’s sister (II-1, 51 years old) accepted our recommendation for hospitalization. After several examinations, we found that the proband’s sister also had the typical tumor symptoms of MEN1, including a pancreatic tumor and a parathyroid tumor. However, due to a force majeure factor, the niece of the proband (III-1) rejected the recommendation to be hospitalized for screening for the disease related to MEN1 in the same year. In addition, any clinical symptoms related to MEN1 had not appeared. It may be because the MEN1-related tumor was not serious or she was not old enough (27 years old). Therefore, we recommended that she be hospitalized at her earliest convenience for screening for the disease related to MEN1. Three years later, the niece of the proband voluntarily was hospitalized because of neck discomfort, and it was found that she had the pancreatic tumor, a parathyroid tumor and an adrenal tumor. The clinical symptoms in this family indicated a *MEN1* gene variant in this Chinese family.

As we all know, the *MEN1* gene plays a role as a growth suppressor in MEN1 tumorigenesis. In the germline, an inactivated variant in the *MEN1* gene on chromosome 11 causes MEN1 syndrome. About 90% of MEN1 cases are usually inherited from affected parents; the other 10% of cases are due to a de novo variant [10]. The loss of heterozygotes at the MENIN site on chromosome 11q13 showed biallelic inactivation of *MEN1* [15]. Knudson’s two-hit model of *MEN1* gene tumorigenesis [3, 4, 16] is supported by the harmful germline variants observed in the MEN1 kindreds and the loss of heterozygosity observed in the tumors of MEN1 patients in a MEN1 family. Since the cloning of the *MEN1* gene in 1997, more than 1,000 families have been reported as MEN1 [17]. Marini et al. reported an analysis of *MEN1* variants in 410 patients’ germlines. It was found that there were 99 different variants, of which 41 were frameshift, 26 missense, 13 nonsense, 11 splice site variants, 4 in-frame small deletions, and 4 large intragenic deletions across one exon [18]. In 2008, Lemos and Thakker conducted a comprehensive analysis of 1336 *MEN1* variants that had been reported in the first decade after the identification of the *MEN1* gene [17]. Paola Concolino et al. reported 208 new germline variants of *MEN1* from 2007 to 2015 [19]. We found the *MEN1* genetic variant c.201delC (p.Ala68Profs*51) to be a new germline variant site in exon 2 that had not yet been reported.

In the *MEN1* gene, the most mutated exons are 2, 9, and 10. In particular, in exons 2 and 10, the most common type of variant is a frameshift [19]. Most of the frameshift and nonsense variants cause protein truncation, resulting in the loss of functional domains, including NLSs located at the C-terminal segment. In the past few years, six new intron variants have been found in MEN1 patients. One of the intron variants (IVS3 + 18 C > T) relates to a c.1546-1547insC variant in a Chinese MEN1 family, which was reported by Zha et al. [20]. Zhiwei Ning et al. [14] found a germline variant of a heterozygous G to A variation at the nucleotide position-1 of intron 5 (c.825-1G > A or IVS5-1G > A) in a MEN1 family.

Interestingly, a very rare situation in this family was that the proband and her daughter were shown to have the homozygous variant in exon 2 when the variant was detected during the first and second times. This result could not be explained by the Mendelian law of inheritance. An animal experiment confirmed that the homozygous variant of *MEN1* could lead to death in rats [21]. Obviously, this “homozygous” variant might have been a mistaken diagnosis.

The first reason that we considered was that the large fragment deletion in the gene, which led to the DNA single strand, was detected twice in the Sanger sequencing. Gross deletions, usually detected by the MLPA technique [22–25], are the rarest kind of *MEN1* variant. The complete *MEN1* gene deletion has been considered by different authors [22, 26, 27]. The first (exons 1–3) [24, 25], central (exons 5–6) [23], and final (exons 8–10) regions of the gene [28] have been described by other gross deletions. Beijers et al. [13] were the first to report on a family with combined germline and somatic mosaicism for MEN1. They used MLPA to analyze the father and found both germline and somatic mosaicism of MEN1. However, the MLPA results showed that there was no large fragment deletion in the genes of the three MEN1 patients in this study.

Faced with this weird “homozygous” variant result, we considered secondly the primer problem. In the former two detections in which we got the suspicious results, we had designed the forward primer in intron 1 and the reverse primer in intron 2 in order to contain all the bases in exon 2. In the third detection, we re-designed both the forward and reverse primers in exon 2 and close to the variant site. We got the gene detection results that not only complied with Mendelian law of inheritance, but also made sense in her genetic family. Thus, we believe that there were some mistakes exist in the first two times gene detections. The possible reason for this situation is that the gene sequence corresponding to primer most probably not compatible with the intronic variant(s) present in this family. In other words, the primer pairs stated for first two gene detections are actually located in regions with a relatively dense variant presence (intron).

We tried to find the variant site or large fragment deletion range in intron 1. However, after too many times experiments, we still can’t accurately detect the variant site in intron. This may be due to the fact that there are relatively dense variants site in the introns, and we cannot design a suitable primer pair to perform the sanger sequence. Since introns have limited meaning in the structure of translation products, they do not code for proteins and will not cause changes in phenotype. Therefore, in this study, we giving up to continue searching for variant site in intron. It could be useful for further scientific research, but useless for the clinical diagnosis and treatment of MEN1 to proband. But in general, it explained the reason for the genetic test error. When faced with this situation again, clinicians could consider the presence of this situation and solve this issue by re-designing the primer closed to the variant site in exon.

There are two limitations for this study. Firstly, the proband has temporarily refused the invasive examinations (including CT or ultrasound-guided needle biopsy) and surgery for any of her tumors, so we could not obtain tumor tissue for further genetic heterozygosity testing, pathological diagnosis, or cell biology testing. Secondly, about the adrenal tumor, we didn’t check the catecholamines because this proband never have hypertension. Thirdly, due to the fact that there are relatively dense variants site in the introns, although we performed too many times experiment try to find out the accurate intron variant site that led to the detection error, it ended in failure. And considering the introns were limited meaningful for the structure of translation products and clinical diagnosis and treatment, we are finally giving up to find the accurate variant site in intron.

This is the first report of a Chinese family with a new MEN1 germline variant in exon 2 (c.201delC (p.Ala68Profs*51)). It can improve the identification of clinical forms of MEN1 and can be used to diagnose the disease at an early stage. In addition, when the detection error present, the clinician should re-design the detection primer close to the variant site could help find the real genetic changes.

## Supplementary information

**Additional file 1**

**Additional file 2**

## Data Availability

Reference sequence for MEN1 (NM_130802.2) is available in the Genbank repository (https://www.ncbi.nlm.nih.gov/nuccore/NM_130802.2). Databases used in this study were Human Gene Mutation Database (HGMD, http://www.hgmd.cf.ac.uk), ClinVar database (https://www.ncbi.nlm.nih.gov/clinvar). We have registered this new germline variant in the National Center for Biotechnology Information. ClinVar; [VCV001064409.1], https://www.ncbi.nlm.nih.gov/clinvar/variation/VCV001064409.1. Data sharing does not apply to this article as no datasets were generated or analyzed during the current study.

## Abbreviations

MEN1: Multiple endocrine neoplasia type 1
AP1: Activator protein 1
LOH: Loss of heterozygosity
CT: Computed Tomography
MRI: Magnetic resonance imaging
EUS: Endoscopic ultrasound
parathyroid ECT: Emission Computed Tomography
EDTA: Ethylenediaminetetraacetic acid
ACMG: American College of Medical Genetics and Genomics
MLPA: Multiplex Ligation-dependent Probe Amplification
INI: Insulin
CPS: C peptide
pro-INS: Pro-insulin
OGTT: Oral glucose tolerance test
Ca^2+^: Calcium
P: Phosphorus
MIBI: 99mTc-stabimidine
COR: Cortisone
DXM: 1-mg overnight dexamethasone
PRL: Prolactin
GH: Growth hormone
IGF-1: Insulin-like growth factor 1
LH: Luteinizing hormone
FSH: Follicle-stimulating hormone
TSH: Thyroid-stimulating hormone
FT4: Free-thyroxine
NET: Neuro-endocrine tumors
GAS-17: Gastrin 17
ZES: Zollinger-Ellison Syndrome
ECL: Entero-Chromaffin-Like
HPT: Hyperparathyroidism
