# Supplementary material for: Multiple endocrine neoplasia type 1: a new germline “homozygous” variant (c.201delC) caused by detection errors
Source: Hered Cancer Clin Pract. 2022 Mar 7;20:10. doi: 10.1186/s13053-022-00216-2 (PMC8900317; doi:10.1186/s13053-022-00216-2)

Supplementary Files

Methods

Laboratory examination

Serum tests for pituitary-related hormones, thyroid and parathyroid hormones, blood glucose, insulin, C-peptide, and bone metabolism-related tests were accomplished in the proband, as well as the related gland function evaluation test. These examinations were performed by the professional Laboratory Medicine Staff in the Medical Laboratory Center, the First Hospital of China Medical University. Pituitary-related hormones levels were measured using IMMULITE2000XPi (SIEMENS AG FWB：SIE, NYSE：SI), thyroid related hormones levels were measured using Cobas 601 analyzer (Roche Diagnostic, Switzerland). Blood glucose, insulin, C-peptide were measured using maglumi4000 (Shenzhen New Industries Biomedical Engineering Co., Ltd, China). Bone metabolism related hormones levels were measured using cobas®8000 e 801 (Roche Diagnostic, Switzerland).

Imaging examination

For a diagnosis of MEN1, the proband completed several imaging examinations, including an ultrasound, CT (computed tomography), MRI (magnetic resonance imaging), endoscopic ultrasound (EUS), and parathyroid ECT (emission computed tomography).

Sanger sequencing

A 5-ml sample of peripheral blood was collected from the proband and each of her six relatives mentioned, anticoagulated with ethylenediaminetetraacetic acid (EDTA),

and stored at –80 °C. Later, whole-genome DNA was extracted. Sanger sequencing was performed on the peripheral blood samples of the six family members using an ABI 3730 sequencer to verify the co-segregation of genotype and phenotype. The DNA sequence of *MEN1* (NM_130802) was queried in the Ensembl Genome Browser, and Primer Premier 6.0 was used to design primers for verification, the gene sequence corresponding to the primer are as follows (Supplementary Fig. 2):

1st times detection: GGGTGGAACCTTAGCGG, AGATGAAATTGGGCTGCA.

2nd times detection: GGGTGGAACCTTAGCGG, CCTCTTTGCAGTTGGGAAAC.

3rd times detection: GACCTGGTGCTCCTTTCCTT, TGTCTATCATCGCCGCCC.

Multiplex ligation-dependent probe amplification (MLPA)

DNA (100 ng) was denatured by heating to 98 °C in a thermocycler. The multiplex ligation-dependent probe amplification (MLPA) probes (probeset name: P017-D1 MEN1, version number: Lot D1-0716, MRC Holland) and buffer were added, and then the samples were left overnight at 60 °C for hybridization. The ligase and ligase buffer were added, and the samples were ligated at 54 °C for 15 min. The ligase was inactivated by heating to 98 °C. PCR primers, dNTPs, and polymerase were added, and the PCR was started. The products were analyzed by capillary electrophoresis.

Coffalyser.Net Server (versions: v.140721.1958) was used for the analysis of sequencing and MLPA reads.

Table 1. The laboratory examination results of hypoglycemia occured.

Items	Results	Normal range
Hypoglycemia occured for the first time		
Intravenous glucose (mmol/l)	2.63	3.89-6.38
Synchronous insulin (mIU/L)	25.86	4.03-23.46
Synchronous c-peptide (pmol/L)	2052.1	99.9-1242.09
Synchronous proinsulin (pg/mL)	253.10	30-180
Insulin release index	0.55	<0.3
Hypoglycemia occured for the second time		
Intravenous glucose (mmol/l)	1.72	3.89-6.38
Synchronous insulin (mIU/L)	16.21	4.03-23.46
Synchronous c-peptide (pmol/L)	1994	99.9-1242.09
Synchronous proinsulin (pg/mL)	264.1	30-180
Insulin release index	0.52	<0.3

Table 1. The proband's serum glucose, insulin (INI), C-peptide (CPS), and pro-insulin (pro-INS) levels were tested when hypoglycemia occured. The insulin release indexes were 0.55 and 0.52 (normal range <0.3) during the twice hypoglycemia, respectively. Glucose was decreased while the insulin level was either in the normal reference range or increased at the time of the hypoglycemia.

Table 2. The results of prolonged OGTT.

	Glucose (mmol/l)	Insulin (mIU/L) (4.03-23.46)	C-peptide (pmol/L) (99.9-1242.09)
0 min	5.16	15.45	1480.8
30 min	10.49	231.3	10060.5
60 min	13.11	217.2	10294.6
120 min	6.57	41.31	7194.8
180 min	3.72	21.52	3098.6
240 min	2.01	17.54	2501
300 min	1.95	15.2	1855

Table 2. The prolonged oral glucose tolerance test (OGTT) results presented hypoglycemia at the 240th and 300th minute with glucose levels of 2.01 mmol/L and 1.95 mmol/L, respectively. The corresponding insulin release indexes were 0.48 and 0.43 (normal range <0.3), respectively.

Figure 1. Multiplex ligation-dependent probe amplification (MLPA) results.

MLPA results showed that the *MEN1* gene was not deleted in the proband (II-3), the proband's sister (II-1), the proband's niece (III-1), and the proband's son (III-2).

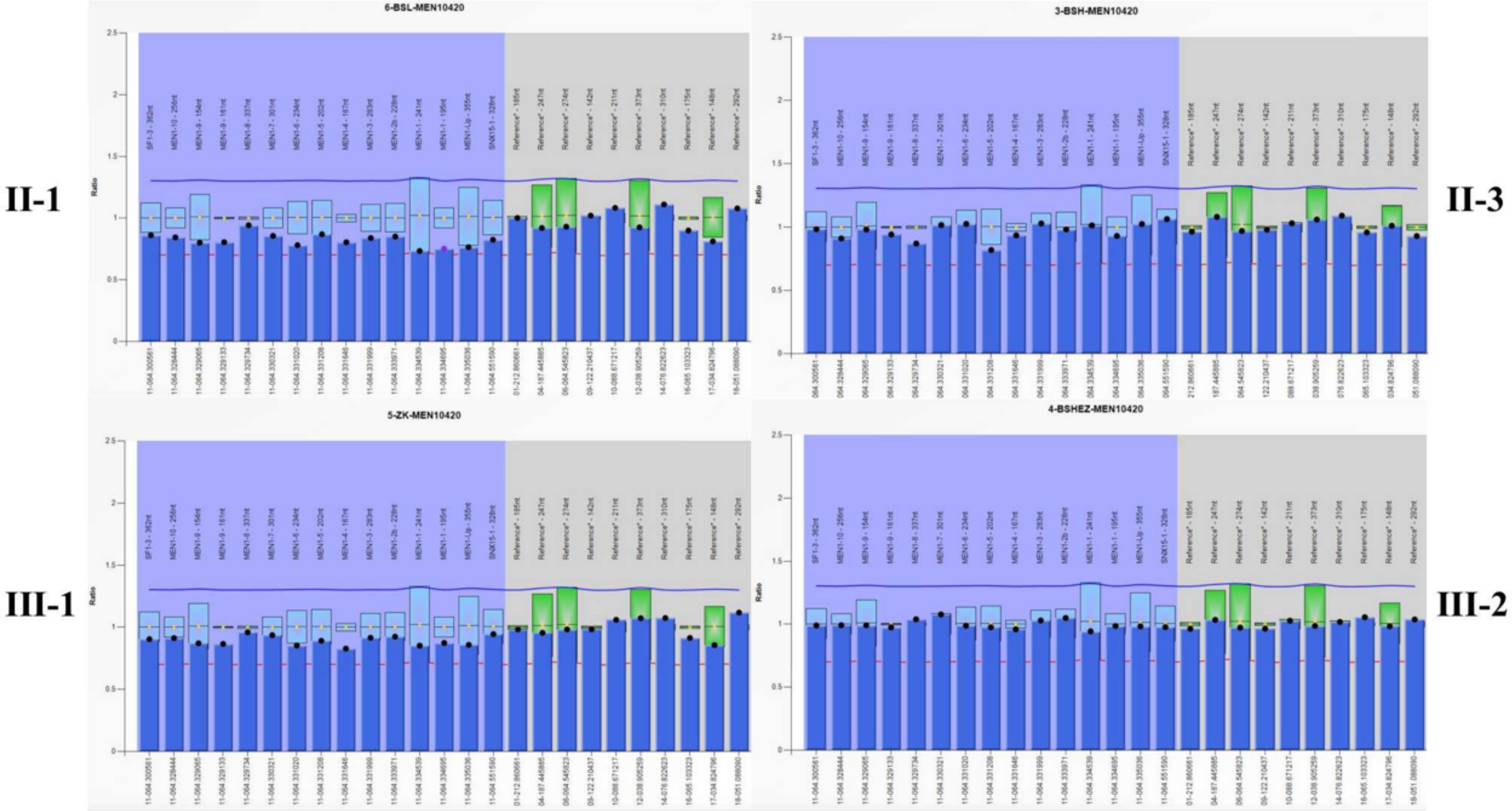

Figure 2. Schematic diagram of the gene sequence corresponding to the primers of the three times genetic detection.

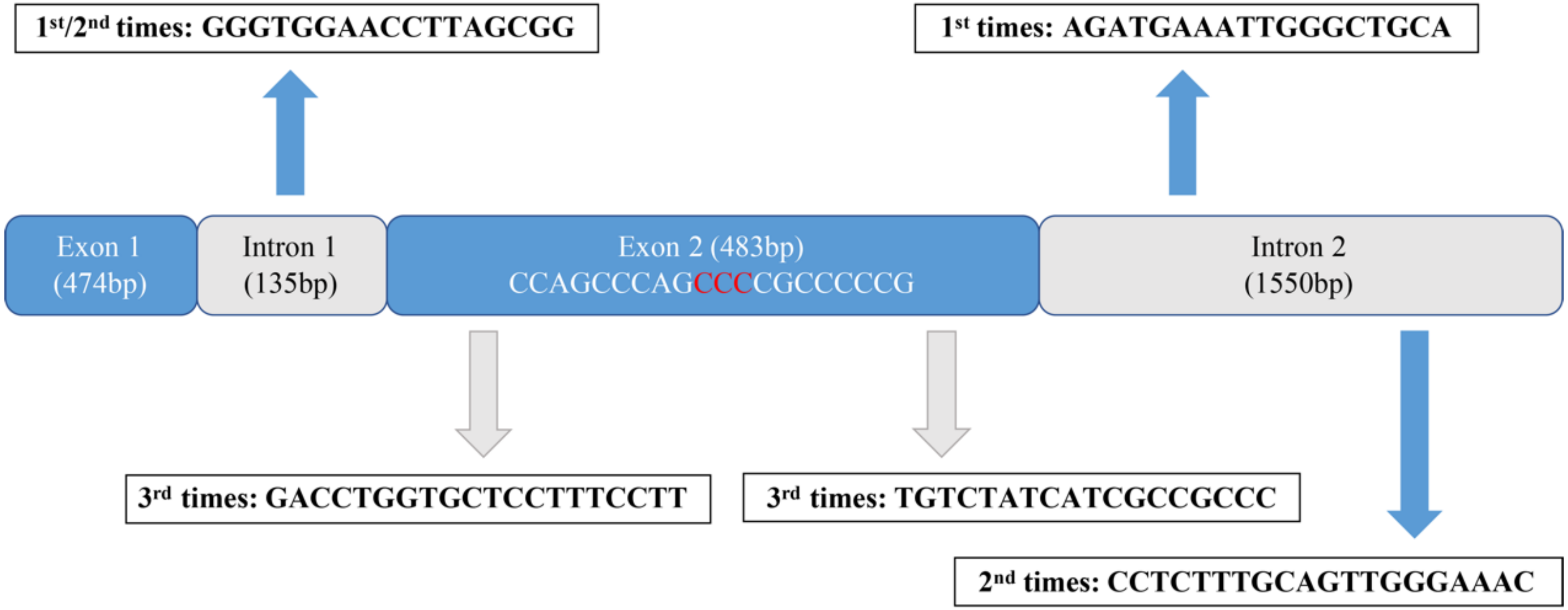

Supplement: Supplementary file 1 — Additional file 1 [file 13053_2022_216_MOESM1_ESM.pdf]
